# Vivisection

**Published:** 1893-02

**Authors:** 


### VIVISECTION.

A prize of $100.00 has been offered by George T. Angell, President of the American Humane Educational Society, for an essay setting forth the best practical plan of decreasing vivisection in the United States.—*Exchange.*

The existence at this day of such a spirit as is indicated in the above item is much to be regretted in the name of progressive and scientific medicine.

These humane and tender-hearted creatures who every now and then raise such a hue and cry about the horrors of vivisection seem to think that those who practice it do so as a sort of ghoulish pastime, and that nothing gives them so much pleasure as witnessing the sufferings of a mutilated cat or guinea-pig. They seem unable to appropriate the idea that the noble men who devote years of their lives in experiments upon the lower animals do so in a spirit of scientific inquiry and in the interest of the science which has for its object the relief of human suffering. It is a fact, to which medical men will bear witness, that the present high position of our science and art, is due in a great measure to knowledge acquired through such experiments by observers whose sole motive was to benefit the human species.

Who, then, is the greater philanthropist, he who seeks to protect some insignificant animals from the so-called cruelty of the vivisectionist, or he who blesses his fellow man by discovering some further means of bringing health and comfort to the sick and suffering ?

It is said that an English lady was once discoursing to the late Sir William Gull upon the cruelty of vivisection; and when she had finished he replied: "Madam, there is no cruelty comparable to ignorance."

In England vivisection has been the subject of frequent agitation for many years. Only recently certain pious brethren in the Church Congress attacked it very vigorously, and we were sorry that they were encouraged by such a distinguished medical man as Mr. Lawson Tait. The defence of it was made by Mr. Victor Horsley, Sir Andrew Clark, Sir James Paget, and others, who claimed that every department of medicine had been promoted by experiments on living animals. Just now the Association for the Advancement of Medicine is preparing to publish a work describing the various experimental researches to which physiology, medicine and surgery are indebted. It is intended to offset the numerous publications of the anti-vivisectionists.

In this connection we may quote from Mr. Huxley, who said, five years ago:

"Unless the fanaticism of philozoic sentiment overpowers the voice of philanthropy, and the love of dogs and cats supersedes that of one's neighbor, the progress of experimental physiology and pathology will, indubitably, in course of time, place medicine and hygiene upon a rational basis. . . . At the present time science, working in the light of clear knowledge, has attacked splenic fever and has beaten it; it is attacking hydrophobia with no mean promise of success; sooner or later it will deal in the same way with diphtheria, typhoid and scarlet fever. To one who has seen half a street swept clear of its children, or has lost his own by these horrible pestilences, passing one's offspring through the fire to Moloch seems humanity compared with the proposal to deprive them of half their chances of health and life because of the discomfort to dogs and cats, rabbits and frogs, which may be involved in the search for means of guarding them."

